# Em defesa da caminhada e do uso de bicicleta como deslocamento no Brasil

**DOI:** 10.1590/0102-311XPT099324

**Published:** 2025-02-28

**Authors:** Alex Antonio Florindo, Guilherme Stefano Goulardins, Douglas Roque Andrade, Margarethe Thaisi Garro Knebel, Maria Paula Santos, Pedro Curi Hallal, Jorge Mota

**Affiliations:** 1 Escola de Artes, Ciências e Humanidades, Universidade de São Paulo, São Paulo, Brasil.; 2 Faculdade de Saúde Pública, Universidade de São Paulo, São Paulo, Brasil.; 3 Grupo de Estudos e Pesquisas Epidemiológicas em Atividade Física e Saúde, Universidade de São Paulo, São Paulo, Brasil.; 4 Faculdade de Desporto, Universidade do Porto, Porto, Portugal.; 5 University of Illinois, Champaign, U.S.A.; 6 Laboratório para a Investigação Integrativa e Translacional em Saúde Populacional, Porto, Portugal.

**Keywords:** Mobilidade Ativa, Caminhada, Ciclismo, Promoção da Saúde, Epidemiologia, Active Mobility, Walking, Bicycling, Health Promotion, Epidemiology, Movilidad Activa, Caminata, Ciclismo, Promoción de la Salud, Epidemiología

## Abstract

Este ensaio tem como objetivo discutir a caminhada e o uso da bicicleta como deslocamento na população brasileira. Evidências científicas oriundas principalmente de países de alta renda mostram tanto contribuições para a saúde das pessoas, apontando que essas atividades físicas podem compor parte importante do cotidiano e contribuem na prevenção de doenças, como para a saúde das cidades, com a diminuição da poluição do ar e sonora e dos problemas provocados pelo excesso de veículos automotores. Discutimos as formas de mensuração em inquéritos nacionais e as novas tecnologias que vêm sendo utilizadas. Análises de tendência mostram uma queda nesse tipo de atividade física entre adultos que vivem nas capitais brasileiras, mas como a caminhada e o uso da bicicleta são analisadas conjuntamente, as interpretações ficam prejudicadas, limitando também o estudo de fatores associados aos diferentes tipos de deslocamentos. Mostramos que algumas capitais brasileiras estão evoluindo no aumento de estruturas ambientais, mas indicadores populacionais mostram que grupos de menor nível socioeconômico têm menos acessos. Discutimos o quanto essas atividades ainda são praticadas por necessidade e sem possibilidades de escolhas no Brasil, onde os custos do transporte ainda são altos e os ambientes, iníquos. No entanto, como a maioria dos estudos no Brasil são transversais, a avaliação dos possíveis efeitos na saúde e a influência de variáveis sociais e de mudanças ambientais nesse comportamento fica prejudicada. Novos inquéritos populacionais e estudos longitudinais que embasem políticas são essenciais para a promoção da caminhada e do uso da bicicleta como deslocamento.

## Abstract (en)

This essay aims to discuss walking and cycling as a means of transportation for the Brazilian population. Scientific evidence (mainly from high-income countries) shows their contributions to people’s health, pointing out that these physical activities can constitute an important part of daily life and contribute to preventing diseases and to the health of municipalities by reducing air and noise pollution and the problems due to the excess of motor vehicles. We discuss the forms of measurement in national surveys and the new technologies that have been used. Trend analyses show a decrease in this type of physical activity in adults living in Brazilian capitals. However, analyzing walking and cycling together impairs interpretations and limits the study of factors associated with different types of commuting. We show that some Brazilian capitals are increasing their environmental structures, but population indicators show that groups of lower socioeconomic levels have less access to the. We discuss how much these activities are still practiced out of necessity − rather than out of choice − in Brazil due to transportation costs that remain high and unequal environments. However, most studies in Brazil being cross-sectional impairs the evaluation of possible effects on health and the influence of social variables and environmental changes on this behavior. New population surveys and longitudinal studies that support policies are essential to promote walking and cycling as a means of transportation.

## Abstract (es)

Este ensayo tiene como objetivo discutir la caminata y el uso de la bicicleta como medio de desplazamiento en la población brasileña. La evidencia científica de países principalmente de altos ingresos muestra contribuciones tanto a la salud de las personas, señalando que estas actividades físicas pueden ser una parte importante de la vida diaria y ayudar en la prevención de enfermedades, como a la salud de las ciudades, mediante la reducción de la contaminación atmosférica y acústica y los problemas causados por el exceso de vehículos de motor. Se discuten las formas de medición en las encuestas nacionales y las nuevas tecnologías que se han utilizado. Los análisis de tendencias muestran una caída en este tipo de actividad física entre los adultos que viven en las capitales brasileñas, pero a medida que se analizan en conjunto la caminata y el uso de la bicicleta, las interpretaciones se ven afectadas, lo que también limita el estudio de los factores asociados con diferentes tipos de desplazamientos. Algunas capitales brasileñas experimentan una evolución en el aumento de las estructuras ambientales, pero los indicadores de población muestran que los grupos de menor nivel socioeconómico todavía tienen menos acceso. Se discuten cómo estas actividades en Brasil aún se practican por necesidad y sin elección cuyos costos de transporte siguen siendo altos y los entornos son poco equitativos. La mayoría de los estudios en Brasil son transversales, lo que afecta la evaluación de los posibles efectos sobre la salud y la influencia de las variables sociales y los cambios ambientales en este comportamiento. Las nuevas encuestas de población y los estudios longitudinales que apoyan las políticas son esenciales para fomentar la caminata y el uso de la bicicleta como medio de desplazamiento.

## Introdução

As práticas de atividades físicas no domínio do deslocamento, como as caminhadas e o uso da bicicleta, que representam parte importante da mobilidade ativa, têm o potencial de promover benefícios para a saúde das pessoas e das cidades. Estudos de revisão mostram que a prática de atividades físicas nesse domínio contribui para a prevenção de doenças ^1^, para a diminuição da circulação de veículos motorizados, para a diminuição da poluição do ar e auditiva e para a diminuição da violência no trânsito ^2^. Além disso, um estudo nacional mostrou que os homens que praticam atividades físicas no deslocamento têm maior chance de também praticarem atividades físicas no lazer ^3^. Ressalta-se também que bairros concebidos com foco na facilidade de deslocamento e em áreas de uso misto do solo tendem a promover mais caminhadas, o que aumenta a possibilidade dos indivíduos conhecerem seus vizinhos, confiarem uns nos outros e se envolverem socialmente ^4^.

No entanto, sabe-se que, especialmente nos países de renda média e baixa e nas famílias de renda baixa nos países de alta renda, a atividade física realizada no deslocamento pode se caracterizar mais como uma necessidade do que como uma escolha. Isso ocorre porque os transportes públicos e coletivos, como ônibus, metrôs e trens, têm alto custo financeiro para as famílias ^5^ ^,^ ^6^, o que muitas vezes faz com que as pessoas caminhem ou usem a bicicleta sem possibilidades de escolhas ^7^. De acordo com dados da *Pesquisa de Orçamentos Familiares* 2017/2018 (POF 2017/2018), a despesa média *per capita* com transporte na região Sudeste do Brasil chegou a 49%, contra 18.9% no Norte, 17,4% no Sul, 9,7% no Centro-oeste e 5% no Norte ^8^. Ou seja, transportes ainda têm alto custo e consomem boa parte dos recursos das famílias. Além disso, estudos epidemiológicos conduzidos no Brasil relacionando a mobilidade ativa por meio da caminhada e do uso da bicicleta com saúde ainda são raros, possuem limitações metodológicas e apresentam resultados diversos. Por exemplo, um estudo transversal realizado com dados da *Pesquisa Nacional de Saúde* (PNS) mostrou uma associação direta entre atividade física no deslocamento e transtornos mentais em jovens de 15 a 24 anos e uma relação inversa entre pessoas de 65 anos ou mais ^9^. Porém, um dos resultados do estudo longitudinal *Inquérito de Saúde de São Paulo* (ISA) − Atividade Física e Ambiente, que acompanha adultos residentes na cidade de São Paulo (Brasil) desde 2014/2015, mostrou que a prática de atividades físicas no deslocamento diminui as chances de desenvolvimento da obesidade ^10^.

É importante refletir sobre a caminhada e o uso da bicicleta como deslocamento levando em consideração os pontos até aqui abordados, mas sem perder de vista o potencial desse domínio da atividade física em melhorar a saúde das pessoas e das cidades brasileiras. O exemplo da caminhada é interessante, pois é uma das atividades físicas mais inclusivas, além de poder ser incorporada com mais facilidade nos programas de promoção da saúde ^11^. Por isso acreditamos que caso a mobilidade ativa seja incorporada por meio da caminhada e do uso da bicicleta como um comportamento nas famílias, idosos, adultos, adolescentes e crianças poderiam caminhar mais para irem de suas residências até escolas, supermercados, restaurantes, centros comerciais, usariam menos veículos motorizados e assim estaríamos contribuindo para a promoção da saúde das pessoas e das cidades. Mas para isso, é preciso que as cidades ofereçam condições seguras para pedestres e ciclistas.

É possível conectar esses argumentos ao direito à cidade, que tem recebido maior atenção de profissionais, de pesquisadores e de movimentos sociais para o fortalecimento da promoção da atividade física e da saúde. Ainda que esse termo seja polissêmico, ele se apresenta como um denominador comum da luta social ^12^, como por exemplo, as agendas de direito ao acesso ao lazer, ao esporte, à cultura e à saúde e, especificamente, à atividade física ^13^ ^,^ ^14^.

A partir dessa introdução, o objetivo deste artigo é argumentar sobre os avanços alcançados até o momento, os desafios, e o que precisa evoluir na epidemiologia para a geração de melhores evidências, programas e políticas públicas efetivas em prol da defesa da promoção da mobilidade ativa, tratada neste artigo como a caminhada e o uso da bicicleta como forma de deslocamento.

## Mensuração de atividade física no deslocamento

Primeiro, gostaríamos de discutir as formas de avaliação desse domínio da atividade física nos estudos epidemiológicos. O Questionário Internacional de Atividade Física (IPAQ − *International Physical Activity Questionnaire*) tem sido muito utilizado pelos pesquisadores latino-americanos, principalmente com adultos e idosos. O questionário IPAQ versão longa avalia separadamente a caminhada e o uso da bicicleta para deslocamento. Estudo que avaliou conjuntamente os domínios do lazer e deslocamento em amostra de adultos que viviam na zona leste de São Paulo mostrou indicadores de validade e reprodutibilidade aceitáveis ^15^. Além disso, artigo científico que discutiu o uso do IPAQ em pesquisas realizadas em países da América Latina, principalmente Colômbia e Brasil, mostrou que os domínios do lazer e do deslocamento na versão longa são considerados adequados nas avaliações populacionais, e são diferentes dos módulos de trabalho e de atividades físicas domésticas, que costumam resultar em superestimativas dos níveis de atividade física ^16^.

O questionário do *Sistema de Vigilância de Fatores de Risco e Proteção para Doenças Crônicas por Inquérito Telefônico* (Vigitel), que é aplicado anualmente desde 2006 nas 26 capitais brasileiras mais o Distrito Federal em amostras de adultos, avalia as atividades de deslocamento como caminhada ou uso de bicicleta de forma conjunta, sem separar os dois tipos de atividades físicas ^17^. Acreditamos que essa é uma limitação do instrumento, pois são atividades físicas muito diferentes, podendo afetar tanto as interpretações de análises de tendência temporal, dando a falsa ideia de que ambas estão decrescendo, como os estudos de fatores associados, que são diferentes para caminhada ou uso de bicicleta. Um exemplo são as análises de tendência publicadas recentemente no relatório Vigitel 2024. Existe um decréscimo nas atividades físicas de deslocamento nas capitais brasileiras desde 2009 até 2023 ^17^. Porém, na cidade de São Paulo, estudo que incluiu 1.434 adultos acompanhados numa coorte mostrou que houve aumento da caminhada como deslocamento, mas não houve alteração quando se analisou somente o uso da bicicleta ou ambas as atividades em conjunto, entre os anos de 2014/2015 a 2020/2021 ^18^ ^,^ ^19^ ^,^ ^20^.

Outra pesquisa importante que também mensura formas de mobilidade ativa é a *Origem e Destino* (OD) da Companhia de Transportes Metropolitanos do Estado de São Paulo ^21^. Iniciada na década de 1970 na Região Metropolitana de São Paulo, a última foi realizada no ano de 2017. Em 2025 teremos a divulgação de uma nova pesquisa OD que trará evidências importantes sobre as formas de mobilidade urbana após a pandemia de COVID-19. O questionário utilizado pela OD é interessante, pois foca especificamente nos diferentes tipos de modais usados para transporte na semana em que a avaliação é realizada, mostrando se as pessoas caminharam ou se usaram a bicicleta, se utilizaram veículos automotores, motocicletas, ônibus, metrô ou trens. Porém, o instrumento não avalia comportamentos ou hábitos de atividade física incorporados no cotidiano. Por exemplo, algumas versões dos módulos de atividade física como deslocamento do IPAQ longo avaliam se as pessoas costumam caminhar ou usar a bicicleta como deslocamento regularmente, independentemente se a atividade foi feita ou não na última semana.

Com relação aos adolescentes, temos dados da *Pesquisa Nacional de Saúde do Escolar* (PeNSE), que é um importante inquérito realizado no Brasil a cada três anos e que tem como objetivo monitorar os fatores de risco e proteção à saúde dos adolescentes em idade escolar. A PeNSE envolve amostras representativas de escolas públicas e privadas de todo o país e já foi realizada em 2009, 2012, 2015 e 2019. Em termos de avaliação da atividade física de deslocamento, os estudantes respondem sobre o comportamento na semana anterior à pesquisa, em que sinalizam quantos dias foram e voltaram a pé ou de bicicleta para a escola. A prevalência brasileira de caminhada ou uso da bicicleta como deslocamento para a escola foi 70,6%, 61,7% e 66,7% em 2009, 2012, 2015, respectivamente ^22^. As prevalências foram significativamente maiores entre os meninos em comparação às meninas. A escolaridade materna influenciou esse tipo de atividade física para a escola, de modo que filhos de mães que não completaram o Ensino Fundamental engajados com atividades físicas de deslocamento foram 79,6%, 67% e 74,7% em 2009, 2012, 2015, respectivamente. Por outro lado, as prevalências de caminhada ou uso da bicicleta como deslocamento quando as mães tinham o Ensino Superior completo foram 52,4%, 49,5%, e 49,8%, respectivamente para 2009, 2012, 2015. No Brasil, também há discrepâncias entre a prevalência de atividades físicas de deslocamento dos escolares ao comparar escolas públicas com privadas, sendo as que as prevalências foram em torno de 1,4 vez maior nas escolas públicas nos anos de 2009, 2012 e 2015 ^22^. No entanto, da mesma forma que no questionário Vigitel para adultos, tanto a caminhada como o uso da bicicleta são avaliados conjuntamente na PeNSE. Portanto, o problema das diferenças entre esses dois tipos de atividades físicas persiste.

Além dos questionários, as avaliações por acelerômetros são cada vez mais frequentes em estudos epidemiológicos para avaliar atividades físicas e nos fornecem uma medida direta do número de passos e da intensidade da atividade física. Porém, apesar do número de passos gerar uma avaliação direta da caminhada, não se pode afirmar se foram para deslocamento ou se foram práticas no lazer ^23^. Além disso, esse método é limitado para avaliar a mobilidade ativa por meio do uso de bicicleta. Para finalizar, são equipamentos de alto custo financeiro e que exigem pessoas especializadas para as análises de dados.

O uso de sistemas de posicionamentos globais, ou *global positioning system* (GPS) em inglês, vêm sendo integrados a outros monitores e medidas para a obtenção de informações mais precisas tanto dos deslocamentos como das atividades físicas ^24^. Frequentemente os estudos de concordância do GPS usam como parâmetros a distância percorrida e a velocidade em situações controladas ^25^ ^,^ ^26^. Também investigam a combinação de GPS com acelerômetros. Estudo que procurou diferenciar as atividades de caminhada, corrida, ciclismo e patinação mostrou que a combinação de acelerometria, número de passos e GPS foi capaz de classificar corretamente essas atividades ^27^. A aplicação do GPS para avaliar a atividade física tem sido empregada nas situações de obtenção de informações sobre posicionamento, rotas preferidas e compreensão do ambiente construído ^24^. Por exemplo, dados de GPS e acelerômetros foram integrados com sistemas de informações geográficas para mapear a vizinhança em um estudo que identificou os períodos e os locais das práticas de atividades físicas moderadas a vigorosas entre adolescentes ^28^. Outro estudo com GPS comparou as diferenças de utilização de ciclovias e vias sem segregação do trânsito para pedalar entre ciclistas ^29^. Assim, reconhecidas suas limitações, o GPS levanta informações interessantes e úteis para ajudar a compreender o padrão de atividade física praticada no ambiente, e pode enriquecer metodologicamente os estudos epidemiológicos quando combinado com outras ferramentas. No entanto, podemos esbarrar em questões éticas, como por exemplo o conhecimento de todos os locais de deslocamento das pessoas, além do alto custo financeiro desses equipamentos nos países de baixa e média renda.

Outros métodos vêm sendo utilizados mais recentemente. A captação dos deslocamentos das pessoas com imagens do Google Street View (https://www.google.com/streetview/), que pode definir categorias de usuários ou veículos nas regiões e classificar os modos de transportes observando pedestres, ciclistas, motociclistas, carros, ônibus e caminhões ^30^, bem como o uso de dados de geolocalização de telefones celulares para monitorar deslocamentos, os quais foram muito utilizados durante a pandemia de COVID-19 para avaliar o efeito das ações de *lockdown* nas cidades, permitindo verificar para onde os sujeitos estão se deslocando ^31^ utilizando as tecnologias de localização por imagens ou por telefonia celular. No caso de smartphones mais avançados com acelerômetros, é possível monitorar a caminhada das pessoas. Esses métodos de coleta de dados podem ser promissores futuramente, mas ainda carecem de mais estudos para verificar a viabilidade em países de baixa e média renda, sendo limitados para avaliar as diferentes atividades físicas com mais qualidade e descrever as características das pessoas, já que ainda dependem de dados agregados.

## Fatores associados à atividade física como deslocamento

Passada a discussão dos avanços e das limitações dos métodos de avaliação, é importante discutir os fatores associados às atividades de caminhada e uso da bicicleta como deslocamento. Os dados do Vigitel mostraram que a razão de prevalência das práticas de atividades físicas no lazer nos homens foi maior nos ativos fisicamente como forma de deslocamento, e que os próprios homens e as pessoas mais jovens e de menor nível de escolaridade foram os mais ativos fisicamente nesse domínio ^3^. Os resultados da comparação com os níveis de escolaridade podem estar reforçando fatores de iniquidade relacionados a esse campo da atividade física.

Para reforçar essa questão, apresentamos e comparamos dois resultados diferentes a seguir. Relatório publicado pelo Ministério da Saúde do Brasil, apresentando os dados de tendência temporal do Vigitel que analisou 806.169 adultos brasileiros que viviam nas capitais e no Distrito Federal de 2009 a 2023, mostrou que houve uma queda nas práticas de atividades físicas de deslocamento. As análises conjuntas de caminhada e de uso de bicicleta por pelo menos 150 minutos por semana caíram de 17% em 2008 para 12% em 2023 ^17^, com redução maior entre pessoas de 25 a 34 anos e de pessoas entre 0 a 8 anos de escolaridade.

Discutindo mais amplamente os resultados desse relatório do Vigitel pelas análises dos dados das 26 capitais mais o Distrito Federal, podemos observar que as capitais mais ativas fisicamente no deslocamento no ano de 2023 foram Belém (Pará), Maceió (Alagoas), Macapá (Amapá), Salvador (Bahia) e São Luís (Maranhão). Importante notar que todas são das regiões Norte e Nordeste. Se formos analisar outros indicadores de desenvolvimento nessas cidades, é possível argumentar que esse tipo de atividade física pode estar sendo praticado mais por necessidade e menos por escolha. Por exemplo, ao analisar o Índice de Desenvolvimento Sustentável da Organização das Nações Unidas (ONU) para os municípios brasileiros elaborado pelo Instituto Cidades Saudáveis, observamos que o escore criado para avaliar o Objetivo do Desenvolvimento Sustentável 11: Cidades e Comunidades Sustentáveis, que engloba variáveis como percentual da população de baixa renda com tempo de deslocamento ao trabalho superior a uma hora, mortes no trânsito, população residente em aglomerados subnormais, domicílios em favelas, equipamentos esportivos e percentual da população negra em assentamentos subnormais, observamos que Belém, Maceió, Macapá, Salvador e São Luís tem escores classificados como muito baixos ^32^. Já cidades como Goiânia (Goiás), Palmas (Tocantins) e Campo Grande (Mato Grosso do Sul), que tiveram as prevalências mais baixas de atividades físicas de deslocamento, possuem também escores considerados médios para o mesmo indicador.

Se formos além e analisarmos a relação das proximidades das estruturas cicloviárias de acordo com a renda *per capita* dos domicílios, observamos que as discrepâncias aumentam. Ao comparar o acesso das famílias mais pobres (com renda *per capita* de até meio salário mínimo) com famílias com renda *per capita* superior a três salários mínimos, observamos que em Belém esse acesso é de 25% para os mais pobres contra 44% para as pessoas com melhor renda. Em Maceió, essas diferenças são ainda maiores, com 4% de acesso para os mais pobres e 24% para as pessoas com maior renda. E mesmo em cidades como Fortaleza (Ceará), onde 51% da população tem acesso a rede cicloviária a até menos 300 metros das suas residências, somente 45% da população mais pobre tem acesso contra 74% da população com maior renda. Isso não é diferente em cidades como São Paulo, que mesmo com o aumento de 67% da rede cicloviária entre 2015 a 2020 ^34^, os dados mostram que somente 16% das pessoas mais pobres têm acesso às ciclovias a até 300 metros das suas residências, contra 36% das pessoas com maior renda ^33^.

Esses dados ficam mais evidentes quando analisamos o projeto *Acesso a Oportunidades*, do Instituto de Pesquisa Econômica Aplicada (IPEA) ^35^. Os pesquisadores utilizaram dados do Censo Demográfico, de registros administrativos e de transporte nas cidades para calcular as diferenças entre os mais ricos e mais pobres no acesso aos empregos formais, estabelecimentos de saúde e de educação. Por meio do cálculo da Razão da Palma, que aponta as diferenças entre os 10% mais ricos comparados aos 40% mais pobres, no ano de 2019 foi mostrado que capitais como Goiânia, Belém, Belo Horizonte (Minas Gerais), São Paulo, Curitiba (Paraná), Campo Grande , Porto Alegre (Rio Grande do Sul), Fortaleza e Maceió estão entre as mais desiguais nos acessos caminhando ou utilizando a bicicleta por até 15 minutos para chegar em empregos formais ou nos equipamentos de saúde. Ou seja, a população com maior renda *per capita* tem mais oportunidades de escolhas para as práticas de atividades físicas de deslocamento.

Nesse contexto, é importante citar a pesquisa de Madeira et al. ^36^, que conduziram um estudo transversal de base populacional com amostra de 12.402 adultos e 6.624 idosos em 100 municípios de 23 estados brasileiros para verificar as prevalências e fatores associados às atividades físicas de deslocamento. Um dos resultados encontrados foi que pessoas de cor da pele branca praticaram menos caminhada e usaram menos a bicicleta em comparação com os pretos e pardos. Os autores argumentam que esse resultado pode evidenciar uma desigualdade de renda e pode indicar que esse tipo de atividade é realizada mais por necessidade do que por escolha.

Esses resultados mostram que os governantes precisam investir na melhora da infraestrutura de acesso, principalmente para as populações mais pobres, para que as pessoas tenham mais oportunidades de escolhas por um tipo de transporte sustentável e saudável para a saúde pública e das cidades.

É importante discutir também outros fatores que são desfavoráveis às práticas de atividades físicas como deslocamento. A violência no trânsito é um deles. Relatório do IPEA mostrou que a mortalidade de pedestres no trânsito cresceu no Brasil entre 2009 a 2019. Os próprios autores discutem que esse aumento da mortalidade no trânsito pode ter relação com o aumento da produção e venda de veículos motorizados ^37^. Ressaltamos que a venda de veículos automotores apresentou um crescimento nos últimos quatro anos e, em 2023, registrou aumento de 9,4% em relação ao ano anterior ^38^. Além disso, houve um crescimento de 21,2% nas vendas de motocicletas no mesmo período ^39^. Por outro lado, as vendas de bicicletas tiveram uma queda de 35% no ano de 2022 em relação a 2021 e 15% no ano de 2023, após um crescimento de 50% no ano de 2020 no período da pandemia de COVID-19 ^40^ ^,^ ^41^, mostrando que esse tipo de bem ainda pode ser de alto custo para a população brasileira.

Citamos também inquérito realizado com representantes de organizações não-governamentais e de entidades públicas e privadas que teve como objetivo verificar a opinião sobre quais ações seriam mais efetivas, factíveis e que os mesmos gostariam de ver testadas em modelos computacionais para aumentar a caminhada e o uso da bicicleta em São Paulo. A principal ação relatada foi a diminuição da velocidade de veículos motorizados ^42^.

Portanto, para se aumentar as possibilidades de escolhas por caminhada e uso da bicicleta, além de investimentos em melhor acesso ao ambiente construído, é importante que tenhamos políticas que combatam à violência no trânsito e que deixem de priorizar o transporte individual por meio de veículos motorizados.

Como já abordado anteriormente, apesar dos dados do relatório Vigitel serem importantes por nos fornecerem os resultados por capitais, eles são limitados por avaliarem conjuntamente a caminhada e uso da bicicleta. Acreditamos que esses resultados por capitais e para todo o Brasil podem mudar se analisarmos tais ações separadamente. Dados do estudo longitudinal do ISA − Atividade Física e Ambiente, que está acompanhando pessoas em São Paulo desde 2014/2015 para verificar determinantes ambientais das práticas de atividades físicas no lazer e como deslocamento, mostrou que não houve mudanças nas prevalências de uso da bicicleta ao longo de seis a sete anos, e que esse comportamento foi predominante nos homens e em pessoas mais jovens e com posse de bicicleta ^18^. Além disso, pessoas fisicamente ativas no lazer ou na caminhada como forma de deslocamento tiveram mais chances de usar a bicicleta ^18^. Outro estudo dessa coorte mostrou aumento na prevalência da caminhada como forma de deslocamento utilizando comparações de 2014/2015 para 2020/2021, porém, quando os dados de caminhada e uso de bicicleta foram analisados de forma conjunta nessa mesma amostra, as prevalências permaneceram estáveis ^19^ ^,^ ^20^. Todos esses resultados mostram que as atividades físicas de deslocamento estão associadas às práticas de lazer, já que as caminhadas estão aumentando em cidades como São Paulo, porém o uso da bicicleta como transporte está estagnado em níveis baixos, apesar de todas as políticas de aumento de infraestrutura das ciclovias que está acontecendo em São Paulo e em diversas cidades brasileiras, mas que geralmente não vem acompanhadas com um processo de educação em saúde ou educação no trânsito ou ações, programas e políticas de estímulo para o uso com foco nos grupos mais vulneráveis.

Apesar dos resultados do relatório do Vigitel mostrarem que as práticas de atividades físicas de deslocamento serem mais prevalentes em pessoas com menor nível de escolaridade, é importante ressaltar os resultados das pesquisas que mostraram que as práticas nesse domínio estão associadas com as práticas no lazer e que uma atividade física não anula outra, fato que coloca em dúvida a noção de que as caminhadas e uso de bicicleta como deslocamento são atividades praticadas por necessidade e que poderiam prejudicar as práticas de atividades físicas no lazer. Portanto, acreditamos que sejam necessários estudos mais detalhados sobre os possíveis determinantes pessoais, sociais e ambientais das atividades físicas, separando as caminhadas do uso da bicicleta em diferentes regiões do Brasil.

Para enriquecer a discussão, com base em análises de dados da última pesquisa OD do Metrô no ano de 2017, verificamos que adultos da cidade de São Paulo com maior escolaridade utilizaram mais a caminhada como modo de transporte (Tabela 1).

**Tabela 1:** Frequência de uso de caminhada como modal de transporte segundo níveis de escolaridade no Município de São Paulo, Brasil, pesquisa *Origem e Destino* (OD), 2017 (n = 47,311).

Níveis de escolaridade	Uso da caminhada	
	%	IC95%
Não alfabetizado e Ensino Fundamental I incompleto	16,8	15,6-18,1
Ensino Fundamental I completo e Fundamental II incompleto	18,6	17,4-19,7
Ensino Fundamental II completo e Ensino Médio incompleto	22,9	21,8-24,1
Ensino Médio completo e Ensino Superior incompleto	20,2	19,6-20,8
Ensino Superior completo	23,1	22,5-23,8

Nota: teste de qui-quadrado, valor de p < 0,001.

Outro estudo que comparou dados de diferentes ODs entre 1997 a 2012 mostrou que o uso da bicicleta como modal de transporte está aumentando em classes sociais de maior poder aquisitivo ^43^. Esses resultados podem indicar que tais práticas podem estar se tornando mais uma alternativa e uma opção do que necessidade, pelo menos em alguns subgrupos da população. No entanto, é importante observar também que os resultados do relatório da OD de 2017 mostram que um dos motivos das pessoas caminharem ou usarem a bicicleta é o alto preço dos transportes públicos e coletivos ^21^.

A posse de veículos também está associada à atividade física de deslocamento. Estudo realizado no Distrito de Ermelino Matarazzo, localizado no extremo leste do Município de São Paulo, mostrou que a inatividade física de deslocamento foi associada com posse de veículos automotores individuais em adultos, indicando uma relação de dependência do carro mesmo em populações mais vulneráveis ^44^. É importante citar que, quanto maior a quantidade de veículos automotores, maior a poluição no ar, pior a qualidade do clima e menor é a atividade física. Além disso, o excesso de veículos automotores circulando, principalmente em municípios de pequeno e médio porte que não priorizam a segurança de pedestres e ciclistas, pode estar relacionado com mortalidade por causas externas no trânsito ^45^. Em megacidades como São Paulo, que tem mais de seis milhões de veículos automotores em circulação ^46^, temos que enfrentar essa situação, o que dificulta muito a promoção da mobilidade ativa por meio da caminhada ou uso da bicicleta. A percepção de segurança, tanto relacionada ao trânsito como contra crimes, foi fator associado às práticas de atividades físicas de deslocamento em estudo transversal que utilizou amostra de 99.967 adolescentes brasileiros da PeNSE 2015 ^47^. Novamente, ressaltamos a importância de se melhorar a segurança no trânsito para que as pessoas tenham mais oportunidades de escolhas para caminhar e usar a bicicleta para deslocamentos ^42^.

Outro ponto favorável na promoção da mobilidade ativa por meio da caminhada e do uso da bicicleta é que esse domínio da atividade física está associado com maior uso de transportes públicos e coletivos. Na coorte ISA − Atividade Física e Ambiente, foi mostrado que a caminhada como deslocamento esteve associada com a utilização de transportes coletivos como trens, ônibus e metrôs nos adultos paulistanos que trabalharam durante a pandemia de COVID-19 ^48^. Enfatizamos que tanto a caminhada como o uso da bicicleta para deslocamentos estão associados com atributos ambientais importantes, como a presença de grandes estações de transportes e de ciclovias nas proximidades das residências. Florindo et al. ^49^ mostraram que as pessoas que viviam a até um quilômetro de distância de estações de trens ou metrôs na cidade de São Paulo tinham mais chances de caminhar como forma de deslocamento. Outro resultado importante já mostrado é que pessoas adultas que viviam a até 1.500 metros de estações de trens e metrôs na cidade de São Paulo também tiveram mais chances de usar a bicicleta como forma de deslocamento ^50^.

## Benefícios da caminhada e uso da bicicleta para a saúde das pessoas e das cidades

Evoluindo para mostrar a importância da mobilidade ativa por meio da caminhada e do uso da bicicleta para a saúde pública e saúde urbana, um artigo publicado na primeira série e um dos editoriais publicados na segunda série de desenho urbano, transporte e saúde na revista *The Lancet* discutiu que investir no conceito de cidades compactas ou bairros compactos dentro de grandes cidades, onde conseguimos fazer a maioria das nossas atividades com deslocamentos de até 30 minutos para trabalho, serviços e estudos por meio de transportes públicos e coletivos, investir em aumentar a quantidade de centros comerciais, escolas, terminais de ônibus, estações de trem e metrô a até 15 minutos de caminhada a partir das residências e em ciclovias próximas às residências pode contribuir na prevenção de doenças crônicas e de mortes por traumas, e por isso deve ser uma prioridade dos gestores e tomadores de decisões nas cidades para a melhora da saúde pública ^51^ ^,^ ^52^. Ações como essas podem contribuir para amenizar o problema dos grandes deslocamentos diários. Por exemplo, na cidade de São Paulo, o tempo médio (ida e volta) para se deslocar para a atividade principal foi de 1 hora e 53 minutos no ano de 2023, sendo de 2 horas e 1 minuto para aqueles que utilizaram os transportes públicos e de 1 hora e 57 minutos para os que utilizaram carro. Houve um aumento de 14 minutos em relação ao ano de 2022 e de 29 minutos em relação ao ano de 2021 ^53^.

Estudo de revisão sistemática mostrou que as práticas de caminhadas e do uso da bicicleta para a mobilidade ativa contribuem para aumentar a atividade física como um todo e diminuir diversas doenças crônicas, e os benefícios da promoção dessas atividades são maiores que os riscos em relação à violência no trânsito, pois a promoção da mobilidade ativa está relacionada com diminuição do uso de veículos individuais motorizados e com uso de transportes públicos e coletivos ^1^. Estudo que analisou as relações entre transporte, energia e saúde apontou que a mobilidade ativa por meio da caminhada e do uso da bicicleta tem grande potencial de melhorar a saúde das pessoas e reduzir a poluição do ar ^54^. Em relação aos riscos e benefícios dessas práticas de atividades físicas quanto à poluição do ar, já que as caminhadas e uso de bicicleta quando utilizados como mobilidade ativa são realizadas próximas a locais onde existe grande circulação dos veículos motorizados, pesquisa experimental que avaliou 10 ciclistas antes e depois da utilização da bicicleta em avenida de alta circulação de veículos automotores em São Paulo mostrou que não houve alterações significativas em indicadores metabólicos para doenças inflamatórias ^55^.

Benefícios relacionados com a melhora do convívio e coesão social e empoderamento das cidades para as pessoas também são importantes, pois a promoção da caminhada aumenta a possibilidade dos indivíduos se conhecerem nas suas vizinhanças, confiarem uns nos outros e se envolverem socialmente ^4^.

Para finalizar, a melhora das condições climáticas e as contribuições para atingir as metas de desenvolvimento sustentável da ONU também são benefícios importantes da promoção da mobilidade ativa ^2^ ^,^ ^56^. Portanto, defender a promoção da caminhada e do uso da bicicleta como deslocamento e garantir ações, programas e políticas públicas intersetoriais nessa direção é defender não somente a melhora da saúde coletiva, mas também da saúde das cidades, além da garantia de direitos. Com mais pessoas caminhando ou utilizando a bicicleta, estaremos contribuindo para a prevenção de doenças, para a melhoria da qualidade de vida das pessoas nas cidades, para a diminuição de lesões e mortalidade no trânsito, para diminuição da poluição do ar e sonora e para a promoção do direito à cidade, além de contribuir para atingir as metas do desenvolvimento sustentável da ONU.

## Direções futuras

Para que a promoção da mobilidade ativa por meio da caminhada e do uso da bicicleta aconteça de forma mais efetiva, acreditamos que devemos avançar em alguns aspectos nessa área no Brasil. Por exemplo na acessibilidade, que é um constructo multidimensional e sua avaliação em estudos epidemiológicos é complexa e essencial ^57^. A acessibilidade na mobilidade urbana pode ser entendida de diversas formas, como a existência de estruturas e calçadas de boa qualidade que permitam que todas as pessoas, independentemente de sua condição individual, de idade ou gênero, as utilizem de forma satisfatória para ir e vir de todos os lugares desejados e identifiquem como um direito à cidade e a prática de atividade física, assim como uma garantia de acesso às atividades de lazer, esporte, cultura e saúde. Na cidade de São Paulo já existem leis implementadas na defesa das calçadas de qualidade, por exemplo o Estatuto do Pedestre, que define a infraestrutura para a caminhada e os espaços que constituem as vias terrestres, que incluem as calçadas, as pistas de rolamento, os canteiros centrais e os logradouros públicos, bem como as estruturas que permitem a conexão delas munidas de facilidade e segurança para as travessias de ruas da cidade ^58^. É preciso que essa discussão esteja mais presente em planos e políticas nacionais, como a Política Nacional de Mobilidade Urbana e o Estatuto das Cidades.

Podemos também entender a acessibilidade ao transporte público, que é basal para a mobilidade, observando aspectos como custos, disponibilidades, distâncias e qualidade. Há também o prisma da inclusão social na acessibilidade. Assim, são necessários estudos que avancem no entendimento desse constructo ^57^. Observatórios de custo-zero ou tarifa-zero e seus impactos na caminhada e no uso da bicicleta são importantes. Por exemplo, estudo experimental realizado com adultos da Tasmânia, Austrália, que comparou grupos que receberam e não receberam compensações financeiras das viagens de ônibus mostrou que, após 16 semanas, o grupo que recebeu as compensações aumentou a atividade física como deslocamento ^59^.

Os planos diretores e os planos de mobilidade urbana das cidades devem conter mais explicitamente ações que contribuam para que as populações mais vulneráveis possam ter escolhas e optar pela mobilidade ativa de acordo com o que foi apontado em artigo científico publicado na série de desenho urbano, transporte e saúde da *The Lancet* ^60^. Esses documentos devem ser mais discutidos no âmbito da saúde pública para que possamos ter métricas mais claras com indicadores populacionais do quanto se deseja aumentar de pessoas caminhando e usando as bicicletas por meio de ações como, por exemplo, construção de calçadas ou de ciclovias, e também o quanto essas ações podem contribuir na prevenção de doenças.

O aumento na quantidade de estudos abordando as contribuições da caminhada ou uso da bicicleta nos níveis de atividade física, o foco em grupos mais vulneráveis como idosos, mulheres e pessoas de baixo nível socioeconômico, bem como os possíveis efeitos do aumento da mobilidade ativa nos níveis de poluição auditiva são importantes ^1^. Além disso, é importante investigar possíveis influências dos tempos de deslocamentos das pessoas em variáveis de saúde e comportamentais, como a própria atividade física no tempo de lazer, pois pessoas que gastam mais tempo nos deslocamentos podem ter piores indicadores de saúde e menos tempo livre para praticarem atividades físicas. Quanto aos métodos de avaliação do ambiente, além das macrovariáveis obtidas por meio de georreferenciamento, uma forma direta que cobre certos indicadores de acessibilidade importantes do microambiente para a mobilidade ativa é o método Global Maps ^61^ ^,^ ^62^, que deve ser melhor explorado em países da América Latina pois permite a caracterização e avaliação de equipamentos urbanos no nível de microescala, em que podemos avaliar a qualidade de calçadas (por exemplo, se seguem as normatizações), a qualidade de ciclovias e a presença de faixas de pedestres e de semáforos. Além disso, mais estudos utilizando telefones celulares para monitorar deslocamentos e avaliar atividades físicas devem ser conduzidos.

Outro ponto que precisa avançar é a discussão da segurança e violência e sua influência na escolha pela caminhada ou uso da bicicleta como deslocamento. Diferentes tipos de crimes têm impacto nas escolhas dos modos de transporte ativo. Por exemplo, crimes violentos podem diminuir as chances de caminhar ou andar de bicicleta ^63^. No entanto, essas discussões precisam ser ampliadas em países como o Brasil. As percepções de segurança, quer relacionadas com o trânsito, aos bairros ou com o medo do crime em geral, estão associadas à atividade física e podem ser diferentes em função do gênero e da idade, por exemplo. Estudos demonstram que as percepções dos pais sobre a segurança relacionada ao trânsito e ao crime estão associadas à mobilidade ativa em crianças e adolescentes ^64^, e a percepção de segurança está relacionada à caminhada como deslocamento de adultos que vivem em regiões de baixo nível socioeconômico em São Paulo ^65^.

Precisamos ter avanços nos sistemas de vigilância. No Brasil, recomendamos que o Vigitel e a PeNSE comecem a avaliar separadamente o uso da bicicleta e a caminhada como atividades físicas de deslocamento. Pesquisadores e lideranças precisam se unir para contribuir no fomento à pesquisa e à transformação em ações em prol da mobilidade ativa por caminhada ou uso da bicicleta. Por exemplo, no Brasil, as organizações sociais e os coletivos têm colaborado com a mobilização e mudança social desejada, como a campanha nacional Calçada Cilada (https://corridaamiga.org/campanhas/calcada-cilada/), realizada desde 2014 pelo Instituto Corrida Amiga com o apoio de diversos parceiros que buscam sensibilizar e engajar a população em favor de cidades acessíveis e caminháveis para todas as pessoas. Outro exemplo é a iniciativa Como Anda? (https://comoanda.org.br/), que apoia a mobilidade a pé e que foi organizada por várias instituições que defendem a mobilidade ativa e campanhas de incidência política, como a realizada pela União Nacional de Ciclistas e parceiros nas eleições para prefeitos, governadores e presidente da República desde 2014 (https://mobilidadenaseleicoes.org.br/nacional/a-campanha/).

Outro ponto relevante são as parcerias entre pesquisadores de diferentes países, de suma importância para a junção de forças para a promoção da mobilidade ativa por meio da caminhada e do uso da bicicleta e na luta contra as prioridades para o transporte individual baseado em veículos automotores e indústria automobilística. A união com gestores e representantes dos movimentos sociais também é extremamente relevante para a elaboração de perguntas relevantes para a pesquisa, o desenvolvimento e a aplicação dos resultados. A promoção da caminhada e do uso da bicicleta como forma de deslocamento não pode ser algo ruim ou obrigatório, porque as pessoas não têm escolhas, mas sim um direito e uma ação essencial para promover a saúde coletiva e a saúde das cidades.

## Data Availability

O acesso a estruturas ambientais que possibilitem escolhas para atividades físicas de deslocamento também é baixo em algumas dessas cidades. Por exemplo, a base MobiliDADOS (https://mobilidados.org.br/), que tem como objetivo monitorar a mobilidade urbana nas 26 capitais e no Distrito Federal, fomentada por diferentes instituições, e que disponibiliza indicadores elaborados a partir de dados abertos disponibilizados pelas Prefeituras, mostrou que a quantidade de pessoas em 2021 que tinham acesso à rede cicloviária a até 300 metros de distância das suas residências era de 7,2% em Maceió, 2% em Macapá e de 2,4% em São Luís. Portanto, indicadores muito baixos se considerarmos que o ambiente construído é importante para possibilitar escolhas. Mas no caso da cidade de Belém, os resultados foram diferentes, pois 31% da sua população tem acesso a alguma rede cicloviária a até 300 metros das residências, mostrando que mesmo em capitais consideradas mais pobres e mais desiguais, existem ações para a melhoria do acesso ao ambiente construído que possibilite oportunidades de escolhas que podem estar surtindo efeito nos indicadores populacionais ^33^.

## Direções futuras

A elaboração de observatórios de cidades saudáveis e sustentáveis, que podem gerar métricas e comparações acessíveis para gestores municipais e estaduais, também são importantes, como o que foi criado a partir da segunda série de desenho urbano, transporte e saúde da *The Lancet*: (https://www.healthysustainablecities.org/).
